# S100A10 and Cancer Hallmarks: Structure, Functions, and its Emerging Role in Ovarian Cancer

**DOI:** 10.3390/ijms19124122

**Published:** 2018-12-19

**Authors:** Tannith M. Noye, Noor A. Lokman, Martin K. Oehler, Carmela Ricciardelli

**Affiliations:** 1 Discipline of Obstetrics and Gynaecology, Adelaide Medical School, Robinson Research Institute, The University of Adelaide, Adelaide 5005, Australia; tannith.noye@adelaide.edu.au (T.M.N.); noor.lokman@adelaide.edu.au (N.A.L.); martin.oehler@adelaide.edu.au (M.K.O.); 2 Department of Gynaecological Oncology, Royal Adelaide Hospital, Adelaide 5000, Australia

**Keywords:** S100A10, annexin A2, plasmin, ovarian cancer, chemotherapy resistance

## Abstract

S100A10, which is also known as p11, is located in the plasma membrane and forms a heterotetramer with annexin A2. The heterotetramer, comprising of two subunits of annexin A2 and S100A10, activates the plasminogen activation pathway, which is involved in cellular repair of normal tissues. Increased expression of annexin A2 and S100A10 in cancer cells leads to increased levels of plasmin—which promotes the degradation of the extracellular matrix—increased angiogenesis, and the invasion of the surrounding organs. Although many studies have investigated the functional role of annexin A2 in cancer cells, including ovarian cancer, S100A10 has been less studied. We recently demonstrated that high stromal annexin A2 and high cytoplasmic S100A10 expression is associated with a 3.4-fold increased risk of progression and 7.9-fold risk of death in ovarian cancer patients. Other studies have linked S100A10 with multidrug resistance in ovarian cancer; however, no functional studies to date have been performed in ovarian cancer cells. This article reviews the current understanding of S100A10 function in cancer with a particular focus on ovarian cancer.

## 1. Introduction

Ovarian cancer is the most lethal gynecological malignancy with a 5-year survival rate of only about 46% [1]. It is estimated for 2018 that worldwide there will be about 295,414 new ovarian cancer cases and 184,799 women will die from this disease [2]. The poor survival rate can be attributed to the fact that ovarian cancer has non-specific symptoms and as a result is often diagnosed at stage 3 or 4. High recurrence rates following treatment and subsequent chemotherapy resistance is another reason [3]. Epithelial ovarian cancers are the most common ovarian malignancies and of that 70% of the subtype are high-grade serous carcinomas. High-grade serous carcinomas have high chemosensitivity following initial treatment with platinum-based therapies, but 75% of patients will relapse and ultimately die from developing chemoresistant disease [4].

Chemotherapy resistance is one of the main reasons for the fatal outcome of ovarian cancer. Thus, discovering and understanding the underlying molecular mechanisms involved in drug resistance is crucial for identifying novel and effective therapeutic targets to be able to improve survival. Resistance mechanisms identified in ovarian cancer include genetic mutations, epigenetic changes, and dysfunctional DNA repair (reviewed in [5,6]). Other identified causes for chemotherapy resistance include upregulation of ATP-binding cassette (ABC) transporters responsible for efflux of cancer therapies [7], activation of cancer stem cells, and epithelial to mesenchymal transition (EMT), as well as alterations to the tumor microenvironment [8,9].

Predicting the response to drug therapies remains a major challenge in ovarian cancer. Currently, there are no predictors of response to first-line chemotherapy in ovarian cancer, and after recurrence, the prediction of the response to second-line chemotherapy is determined empirically from the platinum-free interval (PFI) after the first treatment [4]. Recent studies have used a chemoresponse assay (CRA) to improve patient selection for different chemotherapy treatments [10,11,12,13]. Overall, these studies found that patients with assay-sensitive tissue had improved progression-free survival (PFS) compared to patients with non-sensitive tissue. The inclusion of additional biomarkers that can predict chemotherapy response together with CRAs would enable more effective, individualized patient management and importantly would spare patients from the side effects of ineffective drugs.

Recent studies have linked S100A10 with chemotherapy resistance and poor prognosis in serous ovarian cancer [14,15,16]; however, no functional studies have been performed to date. The aims of this review are to highlight the current understanding of S100A10 function in cancer cells—with a particular focus on ovarian cancer—and to discuss the potential for using S100A10 as a predictive marker and targeting S100A10 to inhibit cancer progression and treatment resistance.

## 2. S100A10 Structure and Function

**Figure 1:**
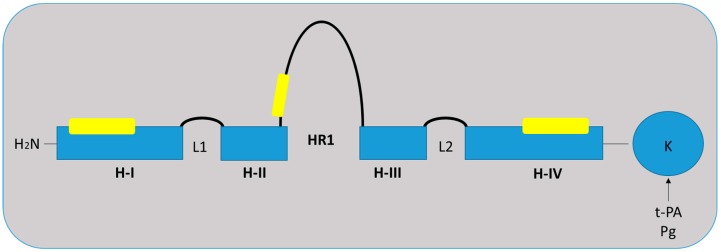
Structure of S100A10 monomer. Each monomer contains four α helical domains H-I, H-II, H-III, and H-IV. Two helical loops L1 and L2 separate H-I and H-II, and H-III and H-1V, respectively. A flexible linker or hinge region (HR1) is also located between H-II and H-III. Binding sites to annexin A2 are located in H-I, HR1, and H-IV, as indicated by the yellow boxes. S100A10 binds both tissue-type plasminogen activator (t-PA) and plasminogen (Pg) via the carboxyl-terminal lysine. Adapted from [32].

S100A10, which is also known as p11 or annexin A2 light chain, belongs to the calcium-binding S100 family, which is characterized by EF-hand calcium-binding motifs [17,18,19]. To date, at least 25 S100 proteins have been identified; the majority are clustered at the chromosome locus 1q21, which is prone to genomic alterations [18]. S100 protein interacts with multiple other proteins and exerts a broad range of cellular functions including (i) phosphorylation; (ii) maintaining cell shape and motility; (iii) calcium homeostasis; (iv) enzyme activity modulation; and (v) transduction pathway signaling [20]. S100 proteins can form both homodimeric and heterodimeric complexes with each other and undergo a conformation change following calcium binding [17]. S100A10 monomers contain four α helical domains (H-I—H-IV) (Figure 1) and are unique from other S100 protein family members as its EF-hands cannot bind to calcium [21]. S100A10 adopts a permanently open conformation comparable to the calcium-bound conformation observed with the other S100 proteins [21]. S100A10 is expressed ubiquitously in the majority of cells and plays a major role in fibrinolysis, wound healing, and angiogenesis [22,23]. Recent studies have shown that S100A10 is important in regulating other physiological processes, including immune cell function [24,25], reproduction [26,27,28], neural cell function [29,30], and heart function [31].

## 3. S100A10 Interaction with Annexin A2

S100A10 is a plasminogen receptor and binds to the cell membrane via its cell surface receptor, annexin A2 [32,33]. Annexin A2 is a soluble monomer in the cytoplasm, and when annexin A2 is sufficiently expressed, it binds to S100A10, forming a stable heterotetramer. Annexin A2 is phosphorylated for conformation changes to occur and translocates the heterotetramer to the cell surface [34]. S100A10 binding to plasminogen results in the activation of plasminogen activators—tissue-type plasminogen activator (t-PA) and urokinase-type plasminogen activator (uPA)—and the conversion of plasminogen to plasmin (reviewed in [32]). S100A10 interacts with annexin A2 to form a heterotetramer, also known as AIIt, which consists of two subunits of annexin A2 and two subunits of S100A10 [35,36]. AIIt activates the plasminogen activation pathway to increase plasmin production in various types of cells [32,37]. Normal endothelial cells utilize S100A10 in the plasminogen activation pathway, converting plasminogen to plasmin, which is vital for fibrinolysis and angiogenesis [22,32]. S100A10 has been shown to regulate up to 90% of plasmin produced in endothelial cells [32]. Plasmin can then degrade fibrin and activate matrix metalloproteinases (MMPs), which in turn promotes degradation of the extracellular matrix (ECM) [38]. S100A10-null mice have been shown to exhibit increased fibrin accumulation [22]. Endothelial cells from S100A10-null mice show a 40% reduction in plasminogen binding and plasmin generation in vitro compared with wild-type mice and exhibit defective angiogenesis [22].

Binding of S100A10 occurs at the annexin A2 N-terminus [39]. This interaction with the annexin A2 N-terminus is required for binding since the removal of this binding leads to the loss of the interaction with S100A10 and results in reduced plasmin production [40]. Several groups have reported that annexin A2 is required to transport S100A10 to the cell surface, which is dependent on the phosphorylation of annexin A2 [34,41,42,43]. S100A10 levels on the cell surface can also be regulated by IFN-*γ* involving annexin A2 via an exosomal secretion pathway [44]. Although earlier studies reported that annexin A2 is a plasminogen receptor [45], more recent studies support that S100A10 rather than annexin A2 is the major plasminogen receptor [46]. It has been proposed that annexin A2 in the heterotetramer plays a role in stabilizing the S100A10 protein and localizing S100A10 to the cell surface. The interaction between S100A10 and annexin A2 is, therefore, thought to protect S100A10 from degradation by the proteasome [21,46,47].

**Figure 2:**
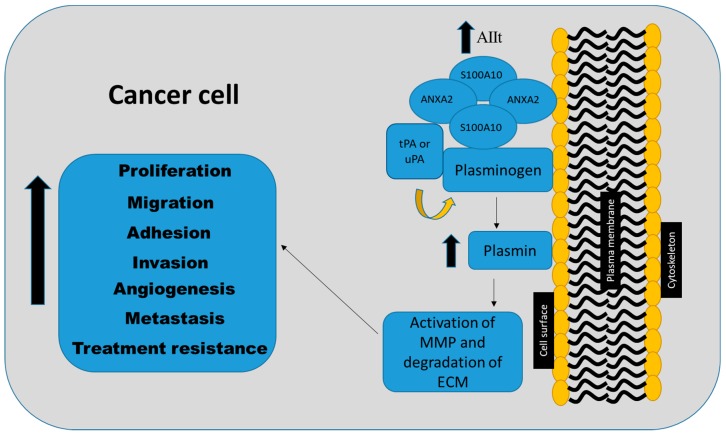
S100A10 plays a key role in regulating pro-tumorigenic processes including proliferation, adhesion, motility, invasion, metastasis, and therapy resistance. The S100A10–annexin A2 heterotetramer (AIIt) acts through the plasminogen activation pathway. AIIt on the cell surface of the plasma membrane activates plasminogen via tissue-type plasminogen activator (t-PA) and urokinase-type plasminogen activator (uPA) and increases the production of plasmin, leading subsequently to the activation of metalloproteinases (MMPs) and the degradation of the extracellular matrix (ECM) proteins, which promote tumor progression and treatment resistance.

In cancer cells, increased annexin A2 and S100A10 expression result in increased plasmin production, which leads to the degradation of the ECM and activation of MMPs, thereby enabling the invasion of surrounding organs or local vasculature (Figure 2) [35,36,37]. Several studies have suggested that the knockdown of annexin A2 concurrently results in the loss of S100A10 [32,48,49,50,51]. Similarly, the loss of S100A10 has been shown to affect both the mRNA and protein levels of annexin A2 [23]. Therefore, it is not known whether the effects observed in many of these studies were mediated by annexin A2 or S100A10. It is likely that both proteins play reciprocal roles in mediating their function in cancer cells.

## 4. Interaction of S100A10 with Other Proteins

In addition to binding annexin A2, S100A10 plays a role in the trafficking of other membrane proteins including sodium channel protein Nav 1.8 [52], potassium channel protein TASK-1 [53], channel proteins TRPV5 and TRPV6 [54], the acid-sensing ion channel ASIC1a [55], and serotonin 5-HT1B receptor [56]. S100A10 is, therefore, thought to play an important role in cell surface presentation of other plasma membrane proteins in addition to annexin A2 [17]. S100A10 functionally acts as a linking protein with the ability to bind transmembrane proteins, thereby aiding the transportation of proteins to the plasma membrane [17]. Protein crystallography has also shown that AIIt interacts with AHNAK, a protein involved in wound repair [57,58], and ARCA3, which is involved in chromatin remodeling [59]. More recent studies have also shown that S100A10 is required for the organization of actin stress fibers and the formation of focal adhesions by HeLa cells via Rac1 signaling [60]. The depletion of S100A10 led to the disruption of stress fiber formation and suppression of Rac1 activation [60].

## 5. S100A10 Expression in Cancers

The expression of S100A10 in cancer has been widely studied and is summarized in Table 1. The role of S100A10 in cancer was first identified in acute promyelocytic leukemia (APL), a subtype characterized by the expression of a fusion protein formed by the fusion of retinoic acid receptor alpha with promyelocytic leukemia (PML-RARα) genes [61]. Patients with APL experience severe bleeding that results from increased plasmin production [61,62]. Increased plasmin levels are due to the upregulation of both annexin A2 and S100A10 in APL cells due to the PML-RARα oncoprotein [46,63]. S100A10 mRNA and protein expression are also upregulated in patients with acute lymphoblastic leukemia (ALL) [64]. S100A10 expression is increased in many other cancers [65,66,67,68,69,70] and is generally associated with a poor prognosis (Table 1). In kidney cancers, *S100A10* expression is 2.5-fold higher than in normal kidney tissue [71]. Non-cancerous resections adjacent to kidney tumors show no expression of S100A10; however, S100A10 is expressed in renal cell carcinoma lesions [72]. *S100A10* expression is increased in melanoma in comparison to levels in normal skin [69]. The expression of S100A10 is also upregulated in basal-type breast cancers [70]. S100A10 is overexpressed in gastric cancers [65,73], precancerous lesions in the stomach [67], and high S100A10 expression is associated with gastric cancer metastasis to the lymph nodes [67]. Overexpression of *S100A10* has also been observed in anaplastic large cell lymphoma [68]. However, reduced S100A10 expression has also been reported in prostate cancer [74] and thyroid carcinoma [75]. Chetcuti et al. reported *S100A10* mRNA expression in prostate cancer tissues but surprisingly found that S100A10 protein is not expressed [74], signifying potential post-translational modifications. S100A10 protein is present in the normal follicular thyroid tissues, but S100A10 expression is reduced in follicular adenoma and follicular thyroid carcinomas [75]. However, increased levels of S100A10 are present in all anaplastic thyroid carcinomas, which is the most aggressive form of thyroid malignancy, suggesting that S100A10 plays a role in the progression of thyroid carcinomas [76]. Tan et al. reported that high cytoplasmic S100A10 expression in advanced stage gallbladder carcinoma is associated with poor prognosis [77]. Overexpression of S100A10 is also associated with poor prognosis in lung carcinoma [78,79] and pancreatic cancer [80]. A recent study identified *S100A10* as one of a three-gene expression signature to independently predict survival of lung adenocarcinoma patients [81]. Similarly, in colorectal carcinomas, increased S100A10 protein is associated with a poor prognosis and reduced overall survival (OS) [82]. Increased *S100A10* expression is independently associated with recurrence in colorectal cancer patients [83].

## 6. Functional Role of S100A10 in Cancer

S100A10 plays a pro-tumorigenic role by regulating proliferation, cell adhesion, migration, invasion, metastasis, and therapy resistance in various malignancies (summarized in Table 2 and Figure 2). Collectively, these studies establish a pro-tumorigenic role for S100A10 as a key contributor in plasmin regulation, tumor progression, and metastasis. 

### 6.1. Proliferation

S100A10 has been linked to play a key role in proliferation in many different types of cancers. Increased cell proliferation of basal-type breast cancer cells is associated with upregulation of S100A10 expression [70]. The knockdown of annexin A2 and concurrent loss of S100A10 expression decreases the cell proliferation of invasive MDA-MB-435S breast cancer cells [50]. The growth of Lewis Lung carcinoma (LLC) and T241 fibrosarcomas is greatly reduced in S100A10 knockout mice compared with wild-type mice [86]. The displacement of S100A10 from annexin A2 attenuates plasminogen activation, impairing colony formation and growth of A549 lung cancer cells [87]. S100A10 has also been shown to bind to Bcl-2-associated death promoter (BAD) protein and adversely affects BAD-induced apoptosis in Chinese hamster ovary (CHO) cells [99]. S100A10 is upregulated by p53 activation, which regulates pro-survival functions in MCF-7 breast cancer cells [85]. Inhibition of hepatic carcinoma, HepG2 cell growth by the microRNA, miR-590-5P is mediated via S100A10 expression [88]. Together annexin A2/S100A10 has also been shown to activate the ERK1/2 and AKT pathways in MM.1S multiple myeloma cells to enhance cell growth [89]. Knockdown of S100A10 by siRNA significantly reduces the proliferation of both HCT-116 and DLD-1 colon cancer cells [82]. A more recent study found that knockdown of S100A10 inhibits the growth of pancreatic cancer cells PANC-1 in immunocompromised NOD/SCID mice [80].

### 6.2. Adhesion

Adhesion of cancer cells is an important phase in the progression of disease. Myrvang and coworkers (2013) were able to show that cell surface S100A10 promotes the adhesion of breast cancer cells to endothelial cells in vitro [91]. S100A10 together with annexin A2 has been shown to regulate the adhesion of leukemia cells [64] and prostate cancer cells to osteoblasts [90]. These findings suggest that S100A10 and annexin A2 may aid the metastatic process by allowing cancer cells to reach the bone marrow.

### 6.3. Migration

S100A10 plays a role in promoting the migration of cancer cells. S100A10 expression promotes the migration of non-small cell lung cancer (NSCLC) A549 cancer cells in vitro [87]. Knockdown of S100A10 by siRNA significantly reduces the migration capacity of two colorectal cancer cell lines, HCT-116 and DLD-1 [82]. Several studies also suggest a direct link between S100A10 expression with the recruitment and migration of macrophages [24,70,86]. S100A10 also plays a critical role in the migration of macrophages to tumor sites and is reported to be a rate-limiting step that controls tumor progression [86].

### 6.4. Invasion

Several studies have shown that S100A10 plays a role in promoting the invasion of cancer cells. Transfection of human HT1080 fibrosarcoma cells with S100A10 antisense oligonucleotides result in a loss of S100A10 protein from the cell surface, decreased plasmin production, and reduced cell invasion [93]. Knockdown of S100A10 by siRNA significantly reduces the invasion capacity of HCT-116 and DLD-1 colorectal cancer cell lines [82]. S100A10 alone in the absence of annexin A2 is crucial for promoting plasmin production and the invasiveness of CCL-22 colorectal cancer cells [92]. A study by Phipps et al. (2011) has also demonstrated that LLC and T241 cells are unable to grow and invade in S100A10-null mice due to the inability to recruit macrophages to the tumor site [86]. The macrophages from S100A10 knockout mice exhibit reduced plasmin-dependent invasion [24]. The displacement of S100A10 from annexin A2 by DLC1, a Rho GTPase-activating protein (RhoGAP) that functions as a tumor suppressor, results in the attenuation of plasminogen activation and impaired invasion of A549 lung cancer cells [87]. The depletion of S100A10 in the kidney (HEK293) and fibroblast (NIH-3T3) cell lines also result in the loss of plasmin production and reduced cell invasiveness [94]. Moreover, a recent study has shown that U937/PR9 and NB4 leukemic cell invasion can be blocked by either annexin A2 or S100A10 antibodies in vitro [63].

### 6.5. Angiogenesis

The process of forming new blood vessels also depends on the presence of S100A10 [86]. Phipps et al. (2011) showed using the S100A10-null mouse model that the density of blood vessels is decreased by over 50% compared with wild-type [86]. S100A10-null macrophages are not able to stimulate angiogenesis and LLC tumor growth in the S100A10-null mice.

### 6.6. Metastasis

As the hallmark of disease progression, metastasis has been shown to be promoted by the presence of S100A10. S100A10 plays an important role in this process, as overexpression of S100A10 in HT1080 fibrosarcoma was shown to increase the lung metastatic burden in mice by 16-fold while the loss of S100A10 reduced the metastatic burden by 3-fold [93]. A more recent study analyzing circulating tumor cells from breast cancer patients reported that *S100A10* is one of the 170 genes activated during intravasation, an important process in the initial stages of metastasis [95].

### 6.7. Therapy Resistance

Increased S100A10 has also been linked to therapy resistance. Treatment with small molecules that inhibit the S100A10–annexin A2 interaction, antibodies against annexin A2 and S100A10, or the knockdown of S100A10 could all increase the sensitivity of NTPL-20 leukemia cells to the chemotherapy drug vincristine [64]. Spijkers-Hagelstein et al. (2013) were able to show that to improve the treatment success of glucocorticoid therapy in ALL, phosphorylation of annexin A2 is required; additionally, this phosphorylation requires S100A10 expression and the absence of both annexin A2 and S100A10 reduces the resistance to treatment in the ALL SEM cell line [96]. COLO-320 colorectal cancer cells that overexpress S100A10 also show reduced sensitivity to oxaplatin [51]. Increased S100A10 has been associated with tamoxifen resistance in MCF-7 breast cancer cells [97] and breast cancer tissues [98]. The mechanisms whereby S100A10 regulates therapy resistance is poorly understood and requires further investigation.

## 7. Role of S100A10 in Ovarian Cancer and Chemotherapy Resistance

**Figure 3:**
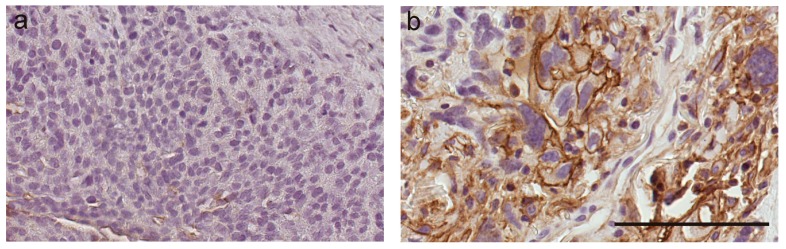
S100A10 immunostaining in matching tissues from a serous ovarian cancer patient at diagnosis (**a**) and recurrence with chemotherapy-resistant disease (**b**). S100A10 expression is increased in tumor tissue at relapse with chemotherapy-resistant disease compared to tumor tissue at diagnosis. S100A10 antibody using citrate buffer retrieval (1/1000, BD Biosciences) [16]. Scale bar = 100 µm.

To date, there have been only three studies that have investigated the expression of S100A10 in ovarian cancer. The study by Gillet et al. (2012), which included 80 serous ovarian cancer patients treated with carboplatin and paclitaxel, found *S100A10* to be one of the 11 signature genes whose expression is involved in multidrug resistance [100]. Another study by Nymoen et al. (2015) found that S100A10 protein expression in ovarian cancer tissues is related to poor chemotherapy response and associated with shorter overall and progression-free survival [15]. In the third study, Lokman et al. (2016) used 13 publicly available ovarian cancer microarray datasets including 722 serous ovarian cancer patients who had received single platinum treatment and 468 patients with combined platinum–taxane treatment [16]. They showed that high mRNA levels of *S100A10* predict reduced OS and that high cytoplasmic S100A10 expression is significantly associated with reduced OS in serous ovarian cancer patients [16]. Moreover, high stromal annexin A2 and high cytoplasmic S100A10 expression in serous ovarian cancer tissues are associated with a 3.4-fold increased risk of progression and a 7.9-fold risk of ovarian cancer death [16]. Our preliminary studies investigating the mechanisms of chemotherapy resistance in ovarian cancer have shown increased S100A10 expression in chemotherapy-resistant disease compared to expression observed in the same patient tissue at diagnosis (Figure 3). Together these findings suggest that S100A10 plays an important role in the progression of serous ovarian cancer and chemotherapy resistance. Future studies are required to investigate further the functional role of S100A10 in ovarian cancer, its usefulness in predicting chemotherapy response, and as a therapeutic target to overcome chemoresistance.

## 8. Strategies to Target S100A10 in Cancer Cells

Different therapeutic strategies have been used to target S100A10 including annexin A2 peptides, S100A10 neutralizing antibodies, small molecule inhibitors, and the vitamin A metabolite all-trans retinoic acid (ATRA). An annexin A2 peptide containing the S100A10 binding site prevents the binding of prostate cancer cells [90] and multiple myeloma cells to osteoblasts [89]. S100A10 antibodies are effective in reducing leukemia cell invasion in vitro [63] and homing of leukemia cells to the bone marrow in vivo [64]. Current studies in our laboratory are investigating the ability of S100A10 antibodies to block serous ovarian cancer motility and invasion.

Using both, a receptor-guided and ligand-guided virtual screening approach has led to the identification of a number of small molecules that inhibit the interaction between annexin A2 and S100A10 [101,102,103,104]. One of these inhibitors 5-benzyl-4-methyl-2-(toluene-4-sulfonylamino)-thiophene-3-carboxylic acid amide that could block the interaction between annexin A2 and S100A10 has recently been shown to inhibit the adhesion of leukemic cells to osteoblasts in vivo and increase the sensitivity of leukemic cells to drugs such as dexamethasone and vincristine in vitro [64]. Further in vitro and in vivo studies are required to determine the effectiveness of these small molecule inhibitors in blocking pro-tumorigenic behavior in a wide range of cancer cells, including ovarian cancer.

ATRA is currently used as a primary treatment for patients with APL [61,62,105]. ATRA has been shown to be beneficial in inducing differentiation and promoting apoptosis of leukemic cells and improving bleeding symptoms by inhibiting plasmin production and decreasing annexin A2 and S100A10 expression [46,106,107,108]. Treatment of APL leukemic cell lines with ATRA causes the rapid loss of both cell surface annexin A2 and S100A10 protein [46,63,109]. Gladwin et al. (2000) also showed that ATRA reduces S100A10 protein levels but not *S100A10* mRNA levels in bronchial epithelial cells (BEAS-2B) [110]. These findings suggest that ATRA exerts its effects to inhibit S100A10 protein levels via a post-translational mechanism. Recent studies further investigating the mechanism whereby ATRA inhibits S100A10 protein levels suggest that ATRA promotes the proteasomal degradation of S100A10 in a ubiquitin-independent manner [111]. ATRA treatment in MCF-7 breast cancer cells reduce S100A10 but not annexin A2 transcript and protein levels, indicating that ATRA can regulate S100A10 levels independently of PML/RARα and annexin A2 [111]. While the effects of ATRA on ovarian cancer cell proliferation and apoptosis have been previously investigated [112], to date no data has been reported on the effects of ATRA on both annexin A2 and S100A10 expression in ovarian cancer cells. Ongoing studies in our laboratory are investigating the ability of ATRA to inhibit cell proliferation and S100A10 expression in a range of serous ovarian cancer cell lines and serous ovarian cancer tissues using an *ex-vivo* explant assay [113]. 

A potential risk of using anti-S100A10 or anti-annexin A2 therapies is the increased risk of thromboembolic events. Previous studies have found increased thrombosis in patients receiving ATRA [114,115] while in other studies the incidence of thrombosis was reported to be lower [116,117]. It will be important to monitor patients treated with anti-S100A10 therapies and consider antithrombotic prophylaxis if they have a high risk of thromboembolic events [118].

## 9. Summary and Conclusions

In conclusion, S100A10 has been shown to play an important role in promoting pro-tumorigenic behavior in many cancers. Emerging evidence shows an important role of the AIIt heterotetramer in the plasminogen activator system in cancer cells and a key role in macrophage migration. The interaction between annexin A2, S100A10, and t-PA mediates the conversion of plasminogen to plasmin, which facilitates the ECM degradation, MMP activation, and angiogenesis, leading to increased cancer cell migration, invasion, and metastasis. S100A10 also plays a significant role in the development of treatment resistance; however, the mechanisms involved are poorly understood and warrant further investigation. A greater understanding of the functional role of S100A10 in ovarian cancer cells could lead to the development of effective strategies to target S100A10 and annexin A2, inhibit progression, and overcome chemotherapy resistance in ovarian cancer patients.

## Figures and Tables

**Table 1:** S100A10 gene and protein expression in cancers.

Cancer	S100A10 Expression	Ref.
Blood	*S100A10* expression is increased on the surface of leukemia cells	[46,63]
	*S100A10* mRNA and protein is upregulated in B-cell acute lymphoblastic leukemia	[64]
Breast	*S100A10* expression is upregulated in basal-type breast cancer	[70]
Colorectal	S100A10 is increased in hereditary polyposis colorectal cancer	[66]
	Increased S100A10 expression is associated with poor prognosis and reduced overall survival in colorectal cancer	[82]
	*S100A10* gene expression is associated with tumor recurrence in colon cancer	[83]
Gallbladder	High cytoplasmic S100A10 expression is associated with poor prognosis	[77]
Kidney	*S100A10* expression is 2.5-fold higher in renal cell carcinoma compared with normal kidney tissue	[71]
	*S100A10* is expressed in renal cell carcinoma and absent in non-cancerous renal tumors	[72]
Lung	Overexpression of S100A10 is associated with poor prognosis	[78,79,84]
Lymphatic	*S100A10* is overexpressed in anaplastic large cell lymphoma	[68]
Pancreas	*S100A10* mRNA and protein is overexpressed in pancreatic cancer and predicts patient outcome	[80]
Prostate	S100A10 expression is lost in prostate cancer tissues	[74]
Skin	*S100A10* expression is increased in melanoma compared with normal skin	[69]
Stomach	*S100A10* is overexpressed in gastric cancer	[65,73]
	S100A10 expression is upregulated in pre-cancerous lesions and associated with gastric cancer metastasis to the lymph node	[67]
Thyroid	S100A10 is overexpressed in anaplastic thyroid carcinomas compared with normal tissues	[76]
	S100A10 expression is decreased in follicular adenomas and thyroid carcinomas	[75]

**Table 2:** The functional roles of S100A10 in cancer cells.

Function	Observation	Ref.
Proliferation	S100A10 is upregulated by p53 activation in breast cancer cells	[85]
	Lewis Lung carcinoma and T241 fibrosarcoma proliferation is inhibited in *S100A10* knockout mice	[86]
	S100A10 is downregulated by a knockdown of annexin A2, which decreases the proliferation of breast cancer cell lines	[50]
	The displacement of S100A10 from annexin A2 attenuates plasminogen activation, impairing colony formation and growth of lung cancer cells	[87]
	Cell growth inhibition by the microRNA miR-590-5P in hepatic carcinoma cells is mediated via S100A10	[88]
	Annexin A2/S100A10 activates the ERK1/2 and AKT pathways in multiple myeloma cells to enhance cell growth	[89]
	S100A10 knockdown reduces proliferation of colon cancer cells	[82]
	S100A10 knockdown inhibits growth of pancreatic cancer cells PANC-1 in immunocompromised NOD/SCID mice	[80]
Adhesion	Annexin A2/S100A10 regulates adhesion of leukemia cells and prostate cancer cells to osteoblasts	[64,90]
	Cell surface S100A10 expression promotes adhesion of breast and prostate cancer cells to endothelial cells in vitro	[90,91]
Migration	S100A10 expression is associated with the recruitment and migration of macrophages	[24,70,86]
	The displacement of S100A10 from annexin A2 attenuates plasminogen activation and impairs the migration of A549 lung cancer cells	[87]
	S100A10 knockdown reduces the migration of colon cancer cells	[82]
Invasion	S100A10 in colon cancer cells is crucial for promoting plasmin production and cell invasiveness	[92]
	S100A10 antibodies inhibit the invasion of acute promyelocytic leukemia cells	[63]
	S100A10 expression in fibrosarcoma cells increases plasmin production and cell invasiveness	[93]
	The displacement of S100A10 from annexin A2 attenuates plasminogen activation and impairs invasion of lung cancer cells	[87]
	Macrophages from S100A10 knockout mice have reduced plasmin-dependent invasion	[24]
	S100A10 depletion in RAS-transformed cell lines (HEK293, NIH-3T3) results in a loss of plasmin production and reduced cell invasiveness	[94]
	siRNA S100A10 reduces invasion of HCT-116 and DLD-1 colon cancer cell lines	[82]
Angiogenesis	S100A10-null mice have reduced blood vessel density compared to wild-type mice	[86]
Metastasis	Loss of S100A10 reduces metastatic burden in the HT1080 fibrosarcoma mouse model	[93]
	Overexpression of S100A10 increases the metastatic burden in the HT1080 fibrosarcoma mouse model	[93]
	S100A10 is one of 170 genes activated during the process of intravasation in breast cancer cells	[95]
Treatment resistance	Disruption of both annexin A2 and S100A10 interactions sensitize leukemia cells to chemotherapy	[64]
	Overexpression of S100A10 reduces the sensitivity of colorectal cancer cells to oxaliplatin	[51]
	Knockdown of S100A10 inhibits annexin A2 phosphorylation and increases sensitivity of acute lymphoblastic leukemia cells to prednisolone	[96]
	S100A10 protein expression is increased in tamoxifen-resistant MCF-7 breast cancer cells and breast cancer tissues	[97,98]

## Abbreviations

AIIt	S100A10-annexin A2 heterotetramer
ALL	acute lymphoblastic leukemia
APL	acute promyelotic leukemia
ABC	ATP-binding cassette
ATRA	all-trans retinoic acid
BAD	Bcl-2-associated death promoter
BEAS-2B	bronchial epithelial cells
CHO	Chinese hamster ovary
CRA	Chemoresponse assay
DLCI	data-link connection identifier
ECM	extracellular matrix
LLC	Lewis Lung Carcinoma
MMP	matrix metalloproteinases
NSCLC	non-small cell lung cancer
PFI	platinum-free interval
PFS	progression-free survival
Pg	plasminogen
PML	promyelocytic leukemia
OS	overall survival
RhoGAP	Rho GTPase-activating protein
t-PA	tissue-type plasminogen activator
uPA	urokinase-type plasminogen activator
